# Role of B7‐H3 in predicting response to neoadjuvant chemotherapy in muscle‐invasive bladder cancer

**DOI:** 10.1002/bco2.418

**Published:** 2024-09-11

**Authors:** Ekamjit S. Deol, Reza Nabavizadeh, Roxane R. Lavoie, Mihai G. Dumbrava, Edlira Horjeti, Prabin Thapa, John C. Cheville, Igor Frank, Fabrice Lucien

**Affiliations:** ^1^ Department of Urology Mayo Clinic Rochester Minnesota USA; ^2^ Division of Experimental Pathology Mayo Clinic Rochester MN; ^3^ Department of Pathology and Laboratory Medicine Mayo Clinic Rochester Minnesota USA

**Keywords:** biomarkers, bladder cancer, cystectomy, neoadjuvant chemotherapy

## Abstract

**Background:**

Neoadjuvant platinum‐based chemotherapy offers a modest survival advantage in muscle‐invasive bladder cancer (MIBC) for patients with pathologic response. B7‐H3 (*CD276*), an immune checkpoint overexpressed in various cancers, including urothelial‐cell carcinoma (UCC), has been associated with chemoresistance and poor oncologic outcomes. We aimed to explore if B7H3 expression on bladder biopsy samples was a predictive biomarker for pathologic response to neoadjuvant platinum‐based chemotherapy.

**Methods:**

This was a retrospective cohort study among MIBC patients receiving neoadjuvant platinum‐based chemotherapy followed by radical cystectomy. All patients underwent routine preoperative biopsy of their tumour. Immunohistochemistry was used to evaluate B7‐H3 expression from pre‐operative specimens. The primary outcome of interest was pathologic complete response (pCR). Statistical analysis included Mann–Whitney *U* test, Fisher's exact test, Kaplan–Meier method, and Cox regression for survival analysis.

**Results:**

Among 87 patients analysed, high B7‐H3 expression was found in 44.8% (*n* = 39) of patients. The median follow‐up periods were similar between the high and low B7‐H3 groups (high expression; 4.29 years [SD 3.04], low expression 3.94 years [SD 3.04], *p* = 0.60). Only 20.5% of patients with high B7‐H3 expression achieved pCR, compared to 41.7% in the low expression group (*p* = 0.04). Cox regression showed no significant differences in recurrence‐free or cancer‐specific survival between the high and low B7‐H3 expression groups (*p* > 0.05).

**Conclusion:**

High B7‐H3 expression is associated with a reduced likelihood of achieving pCR in MIBC patients undergoing neoadjuvant chemotherapy. This suggests B7‐H3's potential as a predictive biomarker for chemotherapy response. Further research is needed to explore the role of B7‐H3 on platinum‐based chemotherapy response in urothelial cancer.

## 1 INTRODUCTION

Platinum‐based drugs are used for the treatment of various cancers including as neoadjuvant therapy for urothelial cancer, due its survival advantage in both pure urothelial carcinoma and histological variants.^1^, ^2^, ^3^ However, the overall survival benefit for neoadjuvant chemotherapy prior to radical cystectomy has only been shown to be ~5%.^2^ Despite guideline recommendations, neoadjuvant chemotherapy, which has been demonstrated to have significant negative side effects, particularly in older and frailer patients, is not widely adopted. Much of the benefit of neoadjuvant chemotherapy occurs if patients experience pathologic complete response (pCR), which represented 28.6% of patients treated in a meta‐analysis by Petrelli et al.^4^, ^5^ Therefore, there is an unmet need to identify predictors of complete pathologic response, allowing clinicians to distinguish between patients who will benefit from neoadjuvant chemotherapy and those who can avoid chemotherapy‐related toxicities and proceed directly to upfront radical cystectomy.

Recently, genetic analysis of bladder cancer tumours has been explored as predictors of pathologic complete response. Several studies have focused on DNA repair pathways including with somatic ERCC2 mutations, and DNA repair genes ATM, RB1, and FANCC predicted pathologic response (presence of either of these mutations was shown to be *p* < 0.001; 87% sensitivity, 100% specificity in Plimack et al.^6^, ^7^). Other studies have focused on molecular subtyping^8^ or detecting the presence of circulating tumour DNA volumes.^9^ Furthermore, an ongoing Phase 2 clinical trial is exploring the potential of gene expression models in predicting response to neoadjuvant chemotherapy and longitudinal survival.^10^

Another promising predictor biomarker of neoadjuvant chemotherapy response is B7‐H3 (*CD276*), an immune checkpoint molecule overexpressed in many cancers, including urothelial carcinoma of the bladder.^11^ Although the molecular mechanisms remain elusive, B7‐H3 expression has been associated with impaired antitumor immunity, poor patient prognosis and resistance to platinum‐based therapy.^12^, ^13^, ^14^, ^15^, ^16^ In a recent study, we showed that high B7‐H3 expression in patients receiving adjuvant chemotherapy following radical cystectomy was associated with worse oncologic outcome (Cancer‐Specific Survival: HR = 2.67, 95% CI 1.18–6.04, *p* = 0.019).^12^ These findings suggest that B7‐H3 may play a crucial role in the response to platinum‐based chemotherapy and could serve as a valuable predictor of neoadjuvant chemotherapy response in patients with urothelial carcinoma of the bladder.

This study aimed to investigate the hypothesis that high B7‐H3 expression is associated with a poorer response to platinum‐based neoadjuvant chemotherapy in patients with muscle‐invasive bladder cancer. To test this hypothesis, we evaluated the relationship between B7‐H3 expression in pre‐treatment biopsies and pathological complete response rates, as well as long‐term oncological outcomes. By examining the potential role of B7‐H3 as a predictive biomarker, we sought to help in identifying patients who may benefit most from neoadjuvant chemotherapy and those who might be better served by alternative treatment strategies.

## 2 METHODS

### 2.1 Patient cohort

This retrospective cohort study was conducted with our institution's prospectively maintained cystectomy registry. Our database was queried for patients who had clinically diagnosed muscle‐invasive disease, received platinum‐based chemotherapy and underwent radical cystectomy between January 1985 and June 2022. Patients were excluded if they did not have tissue slides available for immunohistochemistry staining, had non‐muscle invasive bladder cancer or had previously undergone partial cystectomy prior to radical cystectomy. Patients with both histologic variants and pure urothelial cancer were included, since both previously have been shown to be responsive to neoadjuvant chemotherapy.^17^ Both database querying and retrospective chart review were conducted to abstract clinicopathological variables on age, gender, clinical staging, pathologic staging, neoadjuvant chemotherapy regimen details and oncologic outcomes. The study was conducted in accordance with the Declaration of Helsinki and approved by the Mayo Clinic Institutional Review Board (#20‐000078). The need for informed consent was waived for this study owing to the minimal risk to patient welfare.

### 2.2 Immunohistochemistry staining

The tissue staining protocol was conducted as previously described.^12^ Briefly, tissue slides were independently reviewed by a urologic pathologist expert (JC) to identify tissue blocks positive for urothelial carcinoma. Tissue sectioning (5 μm) was performed at the Pathology Research Core (Mayo Clinic, Rochester, MN). B7‐H3 staining was conducted in our laboratory (D9M2L clone, Cell Signaling Technology, #14058S). Antibody specificity was previously validated using B7‐H3 expressing and B7H3‐knockout RH30 (rhabdomyosarcoma) tumour xenografts.^12^

A standardized H‐score was utilized, ranging from 0 (*no cell membrane expression*) to 300 (*100% cells positive for strong membrane expression*). The H‐score used the following formula: 1 × (% cells weak expression) + 2 × (% cells with moderate expression) + 3 × (% cells strong expression). Expression was only evaluated in tumour cells. H‐score was converted into categorical variables as follows: no or low expression (H‐score ≥ 0 and <100) and high (H‐score ≥ 100).

### 2.3 Publicly available datasets

Two publicly available datasets (GSE87304 and GSE169455) were also analysed for association between B7H3 gene expression and complete pathologic response. GSE87304 was from a retrospective study by Seiler et al and consisted of 309 patients from five centres. GSE169455 was from Sjödahl et al.^18^ and consisted of a retrospective cohort of 149 patients. Both studies conducted whole transcriptome analyses on formalin‐fixed, paraffin‐embedded tumour tissue with GeneChip1 Human Exon 1.0 ST Array (Affymetrix).

### 2.4 Statistical analysis

The primary endpoint was rate of pCR defined as ypT0pN0 at cystectomy. Secondary endpoints included any pathologic downstaging and oncologic outcomes (recurrence free survival and cancer specific survival). All continuous variables were expressed with median (range), whereas categorical variables were expressed as counts and percentages. Between the two B7‐H3 groups (no or low B7‐H3 expression [<100] vs. high expression [≥100]), continuous variables were compared using the Mann–Whitney *U* test, and categorical variables were compared using the Fisher's exact test. The Kaplan–Meier method was used to calculate recurrence‐free, cancer‐specific survival probabilities following cystectomy; differences in survival were assessed using the log‐rank test. For this analysis, patients were censored at the time of last known follow‐up if no event occurred. Univariable Cox regression was performed for recurrence‐free and cancer‐specific survival. Publicly available datasets were normalized by their authors. All statistical tests were two sided with *p* < 0.05 considered statistically significant. Analyses were performed using R version 4.3.1.

## 3 RESULTS

### 3.1 Cohort description

From 1985 to June 2022, our study identified a cohort of 100 patients who underwent radical cystectomy and were treated with neoadjuvant chemotherapy. Among these, tissue blocks suitable for immunohistochemistry analysis were available for 87 patients. Patient characteristics are presented in Table 1. All patients were at a clinical stage of ≥cT2, with a majority being at cT2 (60.9%). The percentage of patients with secondary muscle invasive disease was 16.1% (*n* = 14). Variant histologies were observed in a small subset, including sarcomatoid (*n* = 3), nested (*n* = 1) and plasmacytoid (*n* = 1). Gemcitabine combined with Cisplatin or Carboplatin (GC) was the most used treatment accounting for 73.6% of cases, while methotrexate, vinblastine, adriamycin, and cisplatin (MVAC) was the second most frequent (19.5%). There was no crossover from GC to MVAC regimens. However, four patients transitioned from gemcitabine + cisplatin to gemcitabine + carboplatin. Other treatment regimens included gemcitabine/cisplatin/ifosfamide (*n* = 1), GC followed by pembrolizumab (*n* = 1), cisplatin + etoposide (*n* = 2), cisplatin and vinorelbine (*n* = 1), and taxol + cisplatin (*n* = 1). The average number of chemotherapy cycles did not significantly differ between the no/low and high B7‐H3 expression groups (no/low: 3.90 ± 1.29 vs. high: 3.56 ± 0.64, *p* = 0.15).

**TABLE 1:** Cohort characteristics.

	No or low (*N* = 48)	High (*N* = 39)	Overall (*N* = 87)	*P* value
Age at cystectomy				0.15
Median [min, max]	67.5 [50.0, 84.0]	65.0 [49.0, 80.0]	67.0 [49.0, 84.0]	
Gender				0.72
Female	6 (12.5%)	3 (7.7%)	9 (10.3%)	
Male	42 (87.5%)	36 (92.3%)	78 (89.7%)	
Neoadjuvant chemotherapy regimen				0.19
Gemcitabine + cisplatin/carboplatin	38 (79.2%)	26 (66.7%)	64 (73.6%)	
MVAC	6 (12.5%)	11 (28.2%)	17 (19.5%)	
Other	4 (8.3%)	2 (5.1%)	6 (6.9%)	
Number of NAC cycles				0.042
1	3 (6.3%)	0 (0%)	3 (3.4%)	
2	1 (2.1%)	3 (7.7%)	4 (4.6%)	
3	9 (18.8%)	11 (28.2%)	20 (23.0%)	
3.5	2 (4.2%)	0 (0%)	2 (2.3%)	
4	25 (52.1%)	25 (64.1%)	50 (57.5%)	
5	2 (4.2%)	0 (0%)	2 (2.3%)	
6	5 (10.4%)	0 (0%)	5 (5.7%)	
8	1 (2.1%)	0 (0%)	1 (1.1%)	
Clinical T stage				0.78
cT2	28 (58.3%)	25 (64.1%)	53 (60.9%)	
cT3	17 (35.4%)	11 (28.2%)	28 (32.2%)	
cT4	3 (6.3%)	3 (7.7%)	6 (6.9%)	
Pathologic nodal stage				0.55
pNx	1 (2.1%)	2 (5.1%)	3 (3.4%)	
pN0	39 (81.3%)	26 (66.7%)	65 (74.7%)	
pN1	2 (4.2%)	4 (10.3%)	6 (6.9%)	
pN2	4 (8.3%)	4 (10.3%)	8 (9.2%)	
pN3	2 (4.2%)	3 (7.7%)	5 (5.7%)	
Pathologic tumour stage				0.16
pT0	20 (41.7%)	8 (20.5%)	28 (32.2%)	
pTis/pTa	6 (12.5%)	6 (15.4%)	12 (13.8%)	
pT1	3 (6.3%)	5 (12.8%)	8 (9.2%)	
pT2	8 (16.7%)	8 (20.5%)	16 (18.4%)	
pT3	11 (22.9%)	9 (23.1%)	20 (23.0%)	
pT4	0 (0%)	3 (7.7%)	3 (3.4%)	
Pathologic complete response (ypT0pN0)				0.04
Yes	20 (41.7%)	8 (20.5%)	28 (32.2%)	
No	28 (58.3%)	31 (79.5%)	59 (67.8%)	
B7‐H3 Expression H‐Score				<0.001
Median [min, max]	20.0 [0, 90.0]	180 [100, 300]	70.0 [0, 300]	
Days from NAC end to cystectomy				0.18
Median [min, max]	51.0 [16.0, 381]	42.0 [19.0, 296]	48.0 [16.0, 381]	

### 3.2 B7‐H3 expression and pathologic response rates

Tissue staining revealed high B7‐H3 expression in 39 patients (44.8%), low expression in 39 patients (44.8%) and no expression in 9 patients (10.3%) (Table 1). Pathological downstaging occurred in 18 patients with high B7‐H3 expression versus 31 with low expression (*p* = 0.13). Rates of complete pathologic response (pCR) defined as ypT0pN0 were significantly higher (*p* = 0.04) in the B7‐H3 low group compared to the B7‐H3 high group (*n* = 20 (41.7%) versus *n* = 8 (20.5%); *p* = 0.04). To account for the possibility of occult metastatic disease at the time of surgery, we performed a subgroup analysis excluding seven patients who developed radiographic evidence of distant metastasis within 6 months following surgery. In this subgroup of 80 patients, rates of pCR were 19.4% (*n* = 7) in the high expression group and 45.6% (*n* = 20) in the low expression group (*p* = 0.02) (Table 2).

**TABLE 2:** Rates of pathologic complete response in both B7‐H3 low and high groups.

	Full cohort (*n* = 87)		Cohort without distant recurrence at 6 months (*n* = 80)	
	Not complete response	Complete response	Not complete response	Complete response
B7‐H3 Low	28	20	24	20
B7‐H3 High	32	7	29	7

### 3.3 B7‐H3 expression and oncological outcomes

**FIGURE 1:**
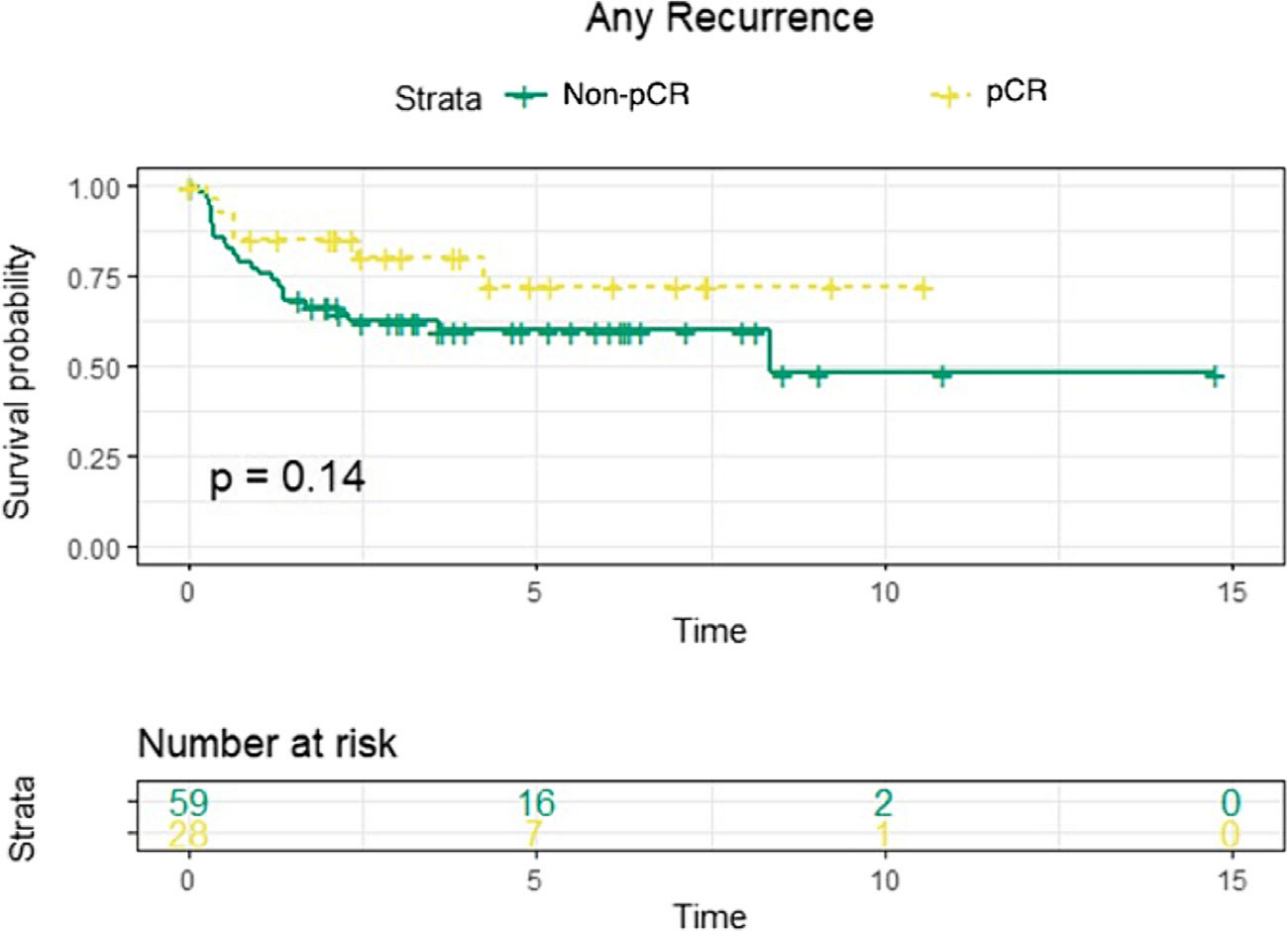
Kaplan–Meier curves of event any recurrence with patients grouped either into pathologic complete response or not complete pathologic complete response.

**FIGURE 2:**
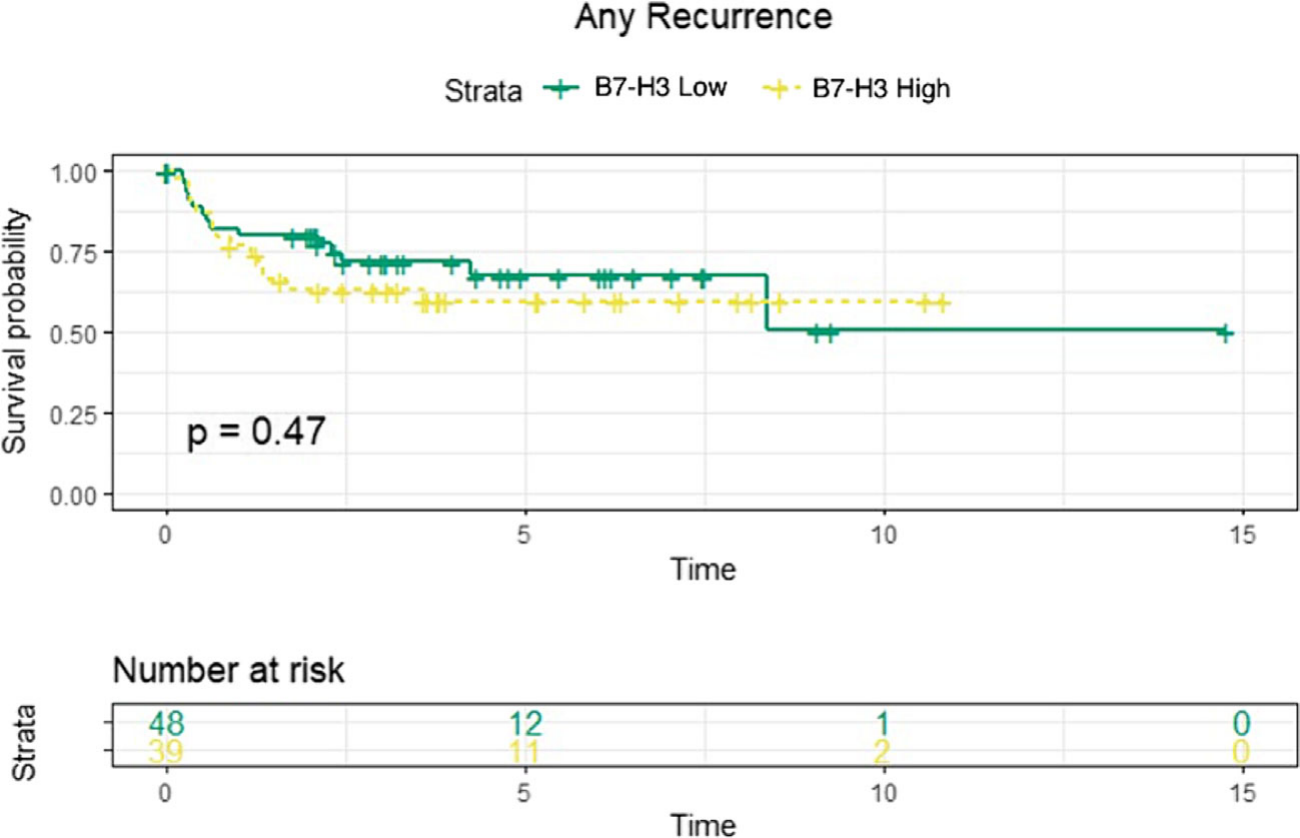
Kaplan–Meier curves of event any recurrence with patients grouped either into low B7H3 expression or high B7H3 expression.

The median follow‐up period was similar between the high and low B7‐H3 expression groups (high expression: 4.29 years, 95% CI: 3.30–5.28; low expression: 3.94 years, 95% CI: 3.06–4.82; *p* = 0.60). Kaplan–Meier analysis revealed no significant differences in recurrence‐free survival between patients with and without pCR (*p* = 0.14; Figure 1) or between the high and low B7‐H3 expression groups (*p* = 0.47; Figure 2). Univariable Cox regression also showed no significant associations between high B7‐H3 expression and recurrence‐free survival (HR: 1.09, 95% CI: 0.45–2.66, *p* = 0.86) or cancer‐specific survival (HR: 1.69, 95% CI: 0.56–5.13, *p* = 0.36). Similarly, pCR was not significantly associated with recurrence‐free survival (HR: 0.51, 95% CI: 0.21–1.25, *p* = 0.14) or cancer‐specific survival (HR: 0.37, 95% CI: 0.12–1.13, *p* = 0.08).

## 4 DISCUSSION

In this exploratory retrospective analysis, we have demonstrated, to our knowledge for the first time, a significant association between B7‐H3 protein expression in urothelial cancer tumours and pathologic complete response in patients undergoing neoadjuvant chemotherapy followed by radical cystectomy for muscle‐invasive bladder cancer. These findings support the hypothesized role of B7‐H3 as a potential biomarker in predicting chemotherapeutic outcomes in bladder cancer, underscoring the need for further investigation. Our results contribute to the growing evidence supporting the role of immunological factors in the response to chemotherapy, particularly in cancers with a known immunogenic nature, such as urothelial carcinoma.

There are several proposed mechanisms by which platinum‐based chemotherapy resistance can occur in urothelial cancer and B7‐H3 may facilitate chemoresistance through several of these mechnisms.^19^ Broadly, the proposed mechanisms include^1^ initial resistance, which limits chemotherapeutic agent accumulation inside cells^2^; direct resistance, facilitated by DNA repair proteins like ERCC1 and systems such as homologous recombination (e.g., BRCA)^3^; subsequent resistance, involving alterations in apoptosis pathways (e.g., changes in p53, BCL‐2)^4^; indirect resistance, which occurs via pathways not directly affected by cisplatin, such as ERBB2/EGFR; and^5^ disruptions in tumour angiogenesis reducing chemotherapeutic delivery.^20^ In vitro studies have shown that B7‐H3 may facilitate platinum‐based chemoresistance through several of the mechanisms described above. Zhang et al. showed that B7‐H3 upregulates DNA repair protein XRCC1 expression in colorectal cancer cells through the PI3K‐AKT pathway.^21^ Ma et al.^22^ and Flem‐Karlsen et al.^15^ demonstrated B7‐H3 contributes to chemoresistance through protection from apoptosis. Ma et al.^22^ showed that B7‐H3 upregulates CD25A expression via the STAT3 signalling pathway in colorectal cancer cells, reducing G2/M phase arrest and enhancing resistance to oxaliplatin. Flem‐Karlsen et al.^15^ revealed that B7‐H3 promotes chemoresistance by activating the p38‐MAPK pathway through DUSP10 inhibition. Additionally, B7‐H3 has been shown to induce a hypoxic tumour microenvironment through aberrant angiogenesis, potentially leading to reduced chemotherapeutic agent delivery and consequently enhancing chemoresistance.^13^, ^20^

The observed relationship between B7‐H3 expression and pathologic complete response, a critical proxy for survival benefit following neoadjuvant chemotherapy, suggests that B7‐H3 may influence urothelial cancer outcomes primarily through a chemotherapy resistance‐dependent mechanism. This hypothesis is supported by our previous study examining adjuvant chemotherapy, which found that patients with high B7‐H3 expression on final radical cystectomy specimens had significantly worse recurrence‐free survival (HR: 2.38, 95% CI 1.06–5.31, *p* = 0.035) and cancer‐specific survival (HR: 2.67, 95% CI 1.18–6.04, *p* = 0.019) compared to those with low or no B7‐H3 expression.^12^ In contrast, a prior study by Xylinas et al.^23^ with only 10% patients receiving adjuvant chemotherapy and no patients receiving neoadjuvant chemotherapy found no significant association between B7‐H3 expression and oncologic outcomes in radical cystectomy patients. Although our study did show a consistent significant association between high B7‐H3 expression and low rates of pCR, the lack of significance on survival analysis likely reflects the modest impact of platinum‐based chemotherapies on survival in the neoadjuvant setting (~5%)^2^ compared to the greater survival benefit of 23% in the adjuvant setting.^24^ The relatively small magnitude of difference in survival, in conjunction with our limited cohort size of 87 patients, likely led to insufficient power to detect significant differences in survival outcomes.

Given our limited sample size, we analysed the association between *CD276* gene expression and pCR rates in two publicly available datasets encompassing a total of 448 patients.^8^, ^18^ Surprisingly, no association was found between *CD276* gene expression and pCR (Tables S1 and S2), contrasting with our tumour tissue analysis. This discrepancy underscores the potential limitations of using gene expression as a surrogate for protein levels. A prior study by Groeneveld et al.,^25^ which conducted a genomic‐proteomic correlation analysis of 23 NMIBC and 40 MIBC tumours, reported a low correlation (0.24) between B7‐H3 gene and protein expression. The divergence between gene expression and protein levels can be attributed to various factors, such as post‐transcriptional modifications, post‐translational modifications, protein degradation rates and temporal differences in expression. Proteins with significant immunological relevance, like B7‐H3, may be subject to additional regulatory mechanisms to fine‐tune their function in the immune response. This complex regulation may account for the observed differences between *CD276* gene expression and B7‐H3 protein levels in relation to pCR in bladder cancer patients, emphasizing the importance of directly assessing protein expression when investigating potential biomarkers.

This study suggests that B7‐H3 inhibition may have a role in dual therapy with platinum‐based agents. B7‐H3 may serve as a target for various therapeutic approaches, including blocking monoclonal antibodies (mAbs), chimeric antigen receptor‐modified T (CAR‐T) cells and combination therapies.^13^ Our study highlights the need for further work in this avenue in wet lab research particularly for combination therapy of B7‐H3 inhibitors with platinum‐based chemotherapy to potentially help improve pCR rates for neoadjuvant chemotherapy.

The interpretation of our findings is subject to several important limitations. The retrospective nature of this study, coupled with a limited cohort size, raises the potential for selection bias. Additionally, being conducted at a single institution, our study necessitates external validation through multicentre retrospective studies. Lastly, although the majority of our cohort was on GC for ≥3 cycles, there were some patients receiving other agents, including in this subset variations in the types of agents, cycle numbers and the interval between completion of neoadjuvant chemotherapy and radical cystectomy.

## 5 CONCLUSION

This study highlights the association between B7‐H3 expression and pathologic complete response in muscle‐invasive bladder cancer patients undergoing neoadjuvant platinum‐based chemotherapy. Our findings suggest that high B7‐H3 expression may be associated with a reduced likelihood of achieving pCR, highlighting its potential role as a predictive biomarker for chemotherapy efficacy. These insights are valuable in the context of the modest overall survival benefits and considerable adverse effects associated with such neoadjuvant chemotherapy, especially in older and more vulnerable patients. While our results point towards the influence of B7‐H3 in chemoresistance rather than direct oncologic outcomes, they open up promising avenues for combination therapy. The possibility of integrating B7‐H3 inhibition with platinum‐based chemotherapy presents an exciting prospect for enhancing therapeutic efficacy and improving patient outcomes. However, there is a need for further investigation to validate the findings of our exploratory cohort.

## AUTHOR CONTRIBUTIONS

FL supervised the study. FL and IF designed the study. JC reviewed tissue specimens and performed H‐scoring. PT abstracted clinical data and supervised statistical analysis. EH and RRL performed tissue staining. ESD, MGD and RN analyzed the data. ESD and FL wrote the manuscript.

## CONFLICT OF INTEREST STATEMENT

The authors have nothing to disclose.

## Supporting information


**Table S1.** GSE87304.
**Table S2**. GSE169455.
