# Supplementary material for: Role of B7‐H3 in predicting response to neoadjuvant chemotherapy in muscle‐invasive bladder cancer
Source: BJUI Compass. 2024 Sep 11;5(11):1052–8. doi: 10.1002/bco2.418 (PMC11557263; doi:10.1002/bco2.418)
Supplement: Supplementary file 1 — Table S1. GSE87304. Table S2. GSE169455. [file BCO2-5-1052-s001.docx]

**Supplementary Table 1**: GSE87304

| **GeneChip1 Human Exon 1.0 ST**  **Array (Affymetrix)**  **Normalized by Authors using single-channel array normalization** | **Pathologic Complete Response (ypT0pN0)**  **(N=90)** | **Non-Pathologic Complete Response**  **(N=209)** | **Mann-Whitney U Test** |
| --- | --- | --- | --- |
| **Median CD276 Gene Expression (IQR)** | 0.51 (0.29) | 0.47 (0.22) | 0.16 |
|  | **Pathologic Complete or Partial Response (ypT0, pTa, pTis) and pN0**  **(N=106)** | **Non-Pathologic Complete or Partial Response**  **(N=193)** | **Mann-Whitney U Test** |
| **Median CD276 Gene Expression (IQR)** | 0.50 (0.28) | 0.47 (0.22) | 0.17 |
|  | **Any Pathologic Downstaging (N=167)** | **No Pathologic Downstaging (N=129)** | **Mann-Whitney U Test** |
| **Median CD276 Gene Expression (IQR)** | 0.49 (0.24) | 0.48 (0.28) | 0.90 |

**Supplementary Table 2**: GSE169455

| **Affymetrix Gene ST 1.0 platform RMA-normalized** | **Pathologic Complete Response (ypT0pN0)**  **(N=48)** | **Non-Pathologic Complete Response**  **(N=101)** | **Mann-Whitney U Test** |
| --- | --- | --- | --- |
| **Median CD276 Gene Expression (IQR)** | 5.48 (0.42) | 5.41 (0.40) | 0.98 |
|  | **Pathologic Complete or Partial Response (ypT0, pTa, pTis) and pN0**  **(N=62)** | **Non-Pathologic Complete or Partial Response**  **(N=87)** | **Mann-Whitney U Test** |
| **Median CD276 Gene Expression (IQR)** | 5.44 (0.43) | 5.41 (0.43) | 0.30 |
|  | **Any Pathologic Downstaging (N=77)** | **No Pathologic Downstaging (N=72)** | **Mann-Whitney U Test** |
| **Median CD276 Gene Expression (IQR)** | 5.49 (0.43) | 5.36 (0.40) | 0.40 |
